# Reward Sensitivity Is Associated with Brain Activity during Erotic Stimulus Processing

**DOI:** 10.1371/journal.pone.0066940

**Published:** 2013-06-28

**Authors:** Victor Costumero, Alfonso Barrós-Loscertales, Juan Carlos Bustamante, Noelia Ventura-Campos, Paola Fuentes, Patricia Rosell-Negre, César Ávila

**Affiliations:** Departamento de Psicología Básica, Clínica y Psicobiologia, Universitat Jaume I, Castellón, Spain; University of Pennsylvania, United States of America

## Abstract

The behavioral approach system (BAS) from Gray’s reinforcement sensitivity theory is a neurobehavioral system involved in the processing of rewarding stimuli that has been related to dopaminergic brain areas. Gray’s theory hypothesizes that the functioning of reward brain areas is modulated by BAS-related traits. To test this hypothesis, we performed an fMRI study where participants viewed erotic and neutral pictures, and cues that predicted their appearance. Forty-five heterosexual men completed the Sensitivity to Reward scale (from the Sensitivity to Punishment and Sensitivity to Reward Questionnaire) to measure BAS-related traits. Results showed that Sensitivity to Reward scores correlated positively with brain activity during reactivity to erotic pictures in the left orbitofrontal cortex, left insula, and right ventral striatum. These results demonstrated a relationship between the BAS and reward sensitivity during the processing of erotic stimuli, filling the gap of previous reports that identified the dopaminergic system as a neural substrate for the BAS during the processing of other rewarding stimuli such as money and food.

## Introduction

The reinforcement sensitivity theory (RST) proposes the existence of a neurobehavioral system involved in the processing of appetitive stimuli [1]–[3]. This system is called the behavioral approach system (BAS) and its primary function is to bring together the individual with biological rewards such as sex and food. The biological substrate of the BAS is thought to comprise brain areas belonging to the dopaminergic reward system [3], which mainly includes subcortical structures such as the ventral tegmental area, substantia nigra, basal ganglia or amygdala, and prefrontal areas such as the orbitofrontal cortex (OFC), medial prefrontal cortex (PFC), and anterior cingulate cortex (ACC) [4], [5].

As claimed by the RST, BAS reactivity increases as a function of the appetitive value of a reward cue or reinforcer, and varies among individuals, resulting in a stable personality trait called reward sensitivity. Behavioral studies have consistently confirmed that individuals with higher scores on measures of reward sensitivity have stronger appetitive conditioning and prefer immediate reward more than low scorers [6], [7]. Accordingly with the RST, previous fMRI studies have shown an association between individual differences in reward sensitivity and brain activity in different BAS-related areas when responding to rewards. For example, Beaver et al. (2006) showed that reward sensitivity was positively associated with activity in the midbrain, ventral striatum (VS), and OFC in response to pictures of appetizing foods [8]. In addition, studies using monetary rewards demonstrated a positive correlation between measures of reward sensitivity and activity in the nucleus accumbens (NAcc), ventral tegmental area, and OFC during processing of reward cues and reinforcers [9]–[11]. In sum, fMRI results have been consistent with the RST, showing that reward sensitivity increases the response of reward brain areas during processing of both reward cues and reinforcers such as money or appetizing foods, leaving an open gap for the processing of sex as a biological reward.

Neural differences in sexual behavior have been less explored in the framework of the RST. Behavioral data have shown that stronger reward sensitivity predisposes a person to be engaged in more sexual experiences, be more curious about sexual topics in the media, and be more sexually excitable [12]–[17]. Sexual behavior is one of the most important goal-directed behaviors essential for the survival of the animal species and is thought to engage brain mechanisms supporting reward processing [18]. A key component of sexual behavior is sexual arousal, defined as physical and psychological readiness to perform sexual behavior [19]. Sexual arousal may be initiated by external stimuli or may be produced by endogenous factors. Recent fMRI studies have used erotic stimuli to study brain areas involved in sexual arousal [19], [20]. These studies showed involvement of BAS-related areas such as the OFC, medial PFC, ACC, VS, and amygdala in sexual arousal. In addition, studies have explored the brain areas involved in processing cues that predict sexual stimuli. For example, activity in the OFC has been demonstrated in response to cues associated with sexual images in participants aware of the contingency [21]. However, no previous studies have analyzed the relationship between individual differences in reward sensitivity and brain activation during anticipation of and reactivity to erotic stimuli.

To study the association between reward sensitivity and the processing of sexual cues and stimuli in more detail, we adapted an event-related fMRI task [22] where erotic and neutral pictures were presented after cues that were 50% or 100% predictive of the erotic stimuli. In line with the RST, we hypothesized stronger activation in BAS-related areas during both the presentation and anticipation of sexual stimuli. In addition, we hypothesized that BAS-related areas involved in the processing of anticipatory cues would show greater activity for cues that were 100% predictive than 50% predictive due to greater contingency between the cue and erotic image. Finally, we expected to observe increased activity in BAS-related areas in participants with high reward sensitivity during the processing of cues and sexual stimuli.

## Methods

### Participants

Forty-five heterosexual men (*M*_age_ = 24.08, *SD* = 3.71, years of education = 13.27±2.93) took part in this study. All participants completed the Sensitivity to Punishment and Sensitivity to Reward Questionnaire (SPSRQ) [23] for a measure of individual differences in reward sensitivity. Three participants were excluded from the personality analyses (see Personality Analysis) because they left more than two items unanswered on the Sensitivity to Reward (SR) scale. The mean score on the SR scale was 12.04 (*SD* = 4.48, range: 2–24, *N* = 42) and scores followed a normal distribution (Kolmogorov-Smirnov test: *D* = .12, *p*>.11); thus, the scores of this sample were similar to those obtained in previous studies [23]–[26]. None of the participants included in the study had a history of head injury with loss of consciousness, currently used psychoactive medications, or were previously or currently diagnosed with DSM-IV Axis I or II disorders, or severe medical or neurological illnesses. Participants provided written informed consent prior to participating in this study and were paid for their participation. The study was approved by the Ethics Committee of the Universitat Jaume I of Castellon.

### Experimental Design and Stimuli

The task used was an adaptation of an earlier study focused on the anticipation of and reactivity to emotionally aversive stimuli [22]. Each trial consisted of a warning cue (X, O, or ?) presented for 1 s and then a fixation point presented for a variable interval of 6, 7, 8, 9, or 10 s, which was then succeeded by a picture presented for 1 s. For appetitive trials, an X cue was always followed by an erotic picture (Ep_100%_). For neutral trials, an O cue was always followed by a neutral picture (Np_100%_). For ambiguous trials, a question mark cue was followed half of the time by an erotic picture (Ep_50%_) and the other half by a neutral picture (Np_50%_). Participants were informed that X and O cues were always followed by erotic pictures and neutral pictures respectively, whereas a question mark cue was followed by either erotic or neutral pictures. Before scanning, participants underwent an exemplary paradigm for 9 min 12 s using a set of erotic and neutral images different from the experimental set. All symbols were white and presented on a black background. Trial order was pseudorandomized with the stipulation that no trial type be presented more than twice in a row. The intertrial interval varied between 6, 7, 8, 9, and 10 s, and was pseudorandomized after both cue and picture presentation. This interval was based on the paradigm of reference in order to optimize jittering for estimation of the hemodynamic response in both the anticipation and response periods [22]. Trial length varied from 14 to 22 s with an average trial length of 18 s.

There were three functional runs, each consisting of eight erotic trials, eight neutral trials, and eight ambiguous trials (totals: 24 positive, 24 neutral, and 24 ambiguous). Each functional scan began with a 10 s black screen, resulting in scan lengths of 7 min, 7 min 15 s, and 7 min 30 s respectively. Runs were randomized among participants. Using a response box (NordicNeuroLab, Bergen, Norway) during the fMRI experiment, participants were instructed to push a single button with their index finger after each cue and each picture to ensure a constant level of attention during picture viewing [27]. Participants were also instructed to respond to 1 s presentations of a fixation cross in isolation during the experimental paradigm (six per run, total = 18) as null events. This stimulus served to maintain participants’ attention to the cue and picture stimuli, and to control for the effects of stimulus response with absent contingencies during reward anticipation and reactivity [28].

During the fMRI experiment, participants viewed 72 pictures (36 erotic and 36 neutral) from the International Affective Picture Set [29] at a resolution of 800×600 pixels with no picture shown more than once. Based on published norms [30], erotic pictures with the highest pleasant valence ratings (*M* = 7.55, *SD* = 1.54) and highest arousal ratings (*M* = 6.97, *SD* = 2.06) comprised the erotic appetitive set, which primarily included photographs of couples and undressed adult women. The selected neutral pictures (e.g., household items) had neutral valence ratings (*M* = 5.03, *SD* = 1.31) and low arousal ratings (*M* = 2.88, *SD* = 2.03). In contrast with other studies involving erotic images [31], [32], we did not use non-erotic pictures of humans for our neutral condition. These stimuli entail diverse variables, such as attractiveness [33], body shape [34], or social valuation [35], that can influence activity in brain areas within the dopaminergic system. Considering how the objective of this study was not to disentangle the specific brain areas responding to sexual arousal but to study the relationship between individual differences in personality and brain activity in response to erotic pictures and cues, we consider pictures of household items better for our neutral condition.

### FMRI Acquisition

Imaging was performed using a 1.5 T Siemens Avanto (Erlangen, Germany) MRI scanner. Functional images were acquired using a gradient-echo T2*-weighted echo-planar MR sequence (TR = 2000 ms, TE = 30 ms, matrix = 64×64×30, voxel size = 3.5 mm^3^, flip angle = 90°, slice gap = .5 mm). We acquired 30 interleaved axial slices oriented parallel to the hippocampus. There were 213 functional volumes for the first run, 218 for the second run, and 214 for the third run. Prior to the functional MR sequences, a high-resolution T1-weighted structural sequence was acquired (TR = 11 ms, TE = 4.9 ms, flip angle = 90°, voxel size = 1×1×1 mm).

### FMRI Analysis

Image processing and statistical analysis were carried out using SPM8 (Wellcome Trust Center for Neuroimaging, London, UK). The first two scans were excluded to allow for equilibration effects. Preprocessing of the functional scans included noise filtering using the ArtRepair toolbox (http://cibsr.stanford.edu/tools/human-brain-project/artrepair-software.html) to repair slice artifacts through interpolation (from before and after scans), slice time correction, realignment to correct for motion-related artifacts, spatial normalization after extracting normalization parameters from the segmentation of each participant’s high-resolution anatomical acquisition (see FMRI Acquisition), and smoothing with an 8-mm (FWHM) Gaussian kernel.

After preprocessing, a general linear model was used to calculate significant hemodynamic changes among the conditions [36]. For the first-level (within-subjects) analyses, each participant’s preprocessed time series were modeled to each condition of interest using the hemodynamic response function and its temporal derivate. Eight regressors were defined for modeling the cues (X, O, and ?), outcomes (Ep_100%_, Np_100%_, Ep_50%_, and Np_50%_), and fixation cross. Furthermore, the six realignment parameters modeling residual motion were also included as regressors of noninterest. Intrinsic autocorrelations were removed via high-pass filter with a cut-off frequency of 1/128 Hz.

Second-level (random-effects) whole-brain voxel-wise analyses were performed to reveal brain activity of the group under the different conditions. For the anticipatory period, one-sample *t*-test analyses were conducted using estimates of BOLD contrasts from the first-level analyses (X>O and ?>O) to obtain BOLD signal differences in response to erotic and ambiguous cues relative to neutral ones. In addition, a paired *t* test was performed to compare differences in the BOLD signal between erotic and ambiguous cues. For the outcome period, a two-way (Condition [erotic, neutral] x Probability [100%, 50%]) repeated-measures ANOVA was performed to compare differences in brain activity regarding the presentation of erotic pictures versus neutral pictures. Additionally, we carried out an exploratory analysis in order to study brain areas that responded to the interaction between condition and probability. Statistical analyses were done at a threshold of *p*<.05 family-wise error (FWE) cluster corrected. The FWE correction was obtained applying a voxel-wise threshold of *p*<.001 uncorrected and a minimum extent threshold of 22 contiguous voxels. The threshold was selected based on Monte Carlo simulations using the Resting-State fMRI Data Analysis Toolkit (REST; http://www.restfmri.net).

### Personality Analysis

Correlation analyses were performed in order to study the relationship between SR scores and brain activity during the anticipation and outcome conditions. Following previous studies of individual differences [11], [37], [38], we analyzed the association between reward sensitivity and brain activity by correlating an individual’s SR scores and the mean value of activity in specific brain areas of interest. Our analysis was restricted to volumes of interest (VOIs) in areas belonging to the dopaminergic system, including the midbrain, striatum, amygdala, medial PFC, OFC, and insula [5], [39]. The peak maximum coordinates of dopaminergic areas that showed significance in the whole-brain voxel-wise analyses were used to define the VOIs. Every VOI consisted in an 8-mm radius sphere centered on the peak voxel. For each participant, the mean BOLD contrast estimates of all active voxels within the VOI were calculated. Finally, these values were included in a partial correlation with SR scores, removing the effect of age. We included age as a covariate because previous evidence has shown that this variable is related to brain activity within the dopaminergic system [40], [41]. The correlation analysis threshold was set to *p*<.05 Bonferroni FWE corrected. Based on this method, we divided the a priori selected threshold of *p*<.05 by the number of tests performed (*k = *9; see Results), which stabilized statistical levels as significant if less than.0055.

### Behavioral Analysis

The median reaction times (RTs) of responses to anticipatory cues and responses to images were separately recorded for each participant to perform behavioral analyses. Paired *t* tests were carried out to study differences among anticipatory cues (X>?, X>O, and ?>O). To study differences during the outcome period, a two-way (Condition [erotic, neutral] x Probability [100%, 50%]) repeated-measures ANOVA was conducted. Finally, in order to study personality effects on RT, we performed partial correlations between SR scores and the RTs for erotic conditions (cues and images), controlling RTs for their respective neutral conditions.

## Results

### Behavioral Results

Behavioral analyses showed slower RTs for ambiguous cues (*M*_?_ = 458.4±116.2 ms) than for both erotic cues (*M*_X_ = 445.22±126.64 ms, *t* = 2.85, *p* = .007) and neutral cues (*M*_O_ = 436.8±139.7 ms, *t* = 3.67, *p* = .001). No differences were found between RTs for erotic and neutral cues (*p*>.05). On the other hand, the analysis of RTs for images (*M*_Ep100%_ = 388.5±128.9, *M*_Np100%_ = 360.2±99.1, *M*_Ep50%_ = 389.6±125, *M*_Np50%_ = 374.8±121.8) showed a main effect of condition, *F*(1, 44) = 11.18, *p = *.001, indicating slower RTs for erotic than neutral images. This result may signify that participants pay more attention to erotic than neutral images. However, these results should be cautiously interpreted given that participants were not instructed to respond as soon as possible but to answer as a measure of their attention, following the paradigm of reference [22]. Finally, no significant correlation was obtained between SR scores and RTs.

### FMRI Results

**Figure 1:**
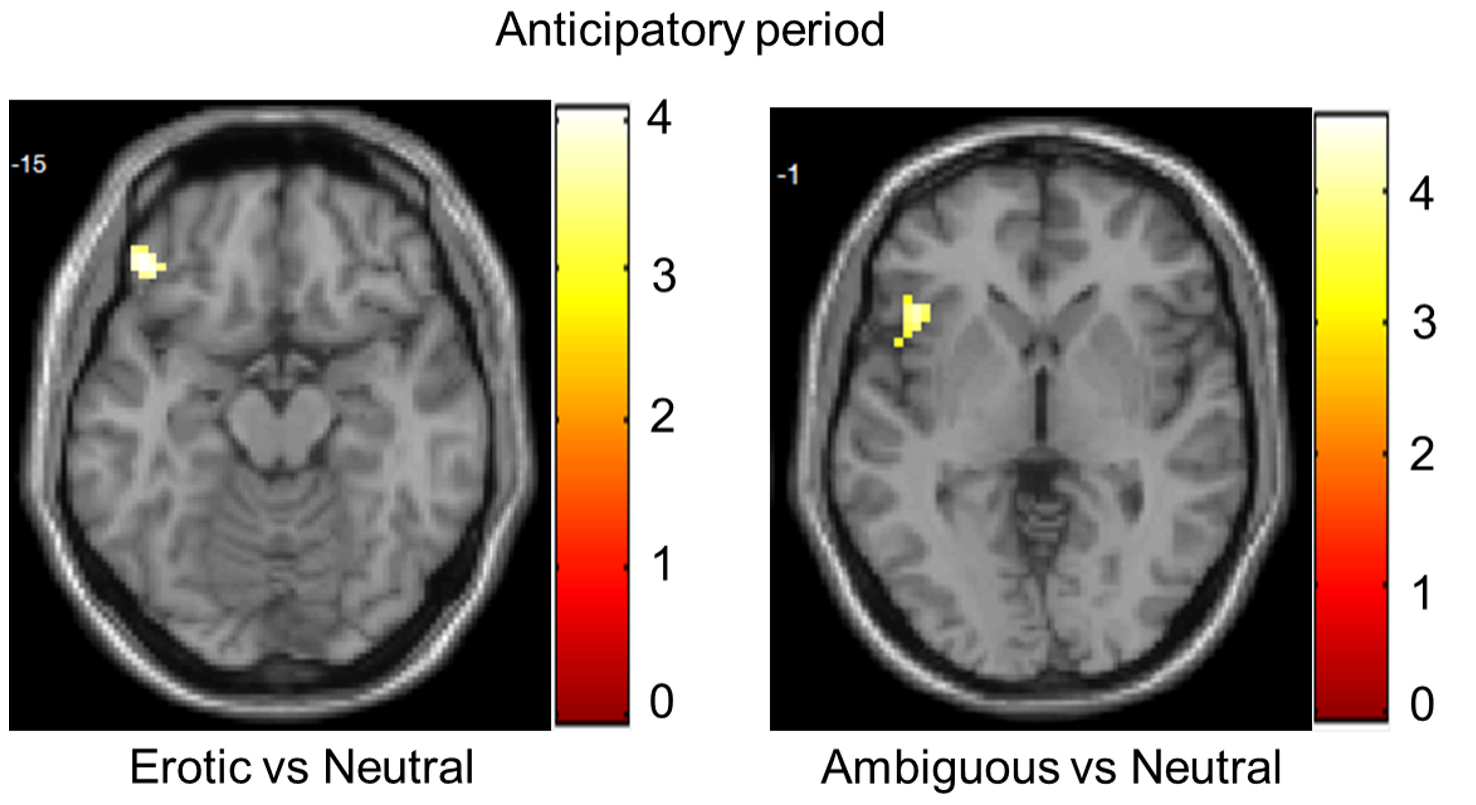
Brain activity during anticipatory cue processing. Images are presented in neurological convention (left is left) and with a threshold at *p*<.05 corrected. The color bars represent the *t* values applicable to the images and the numbers within the images correspond to z MNI coordinates.

Whole-brain analyses showed the involvement of BAS-related areas in both the anticipation of and reactivity toward erotic stimuli. Analyses of the anticipatory period demonstrated enhanced activity in the left OFC (x, y, z: −45, 38, −14; *z* = 3.73, *k* = 25) during the presentation of erotic cues in comparison with neutral cues. Moreover, activity in the left anterior insula (x, y, z: −42, 20, 1; *z* = 4.11, *k* = 25) was related to the presentation of ambiguous cues but not neutral cues (Figure 1). By contrast, no significant differences were found when erotic and ambiguous cues were compared.

**Figure 2:**
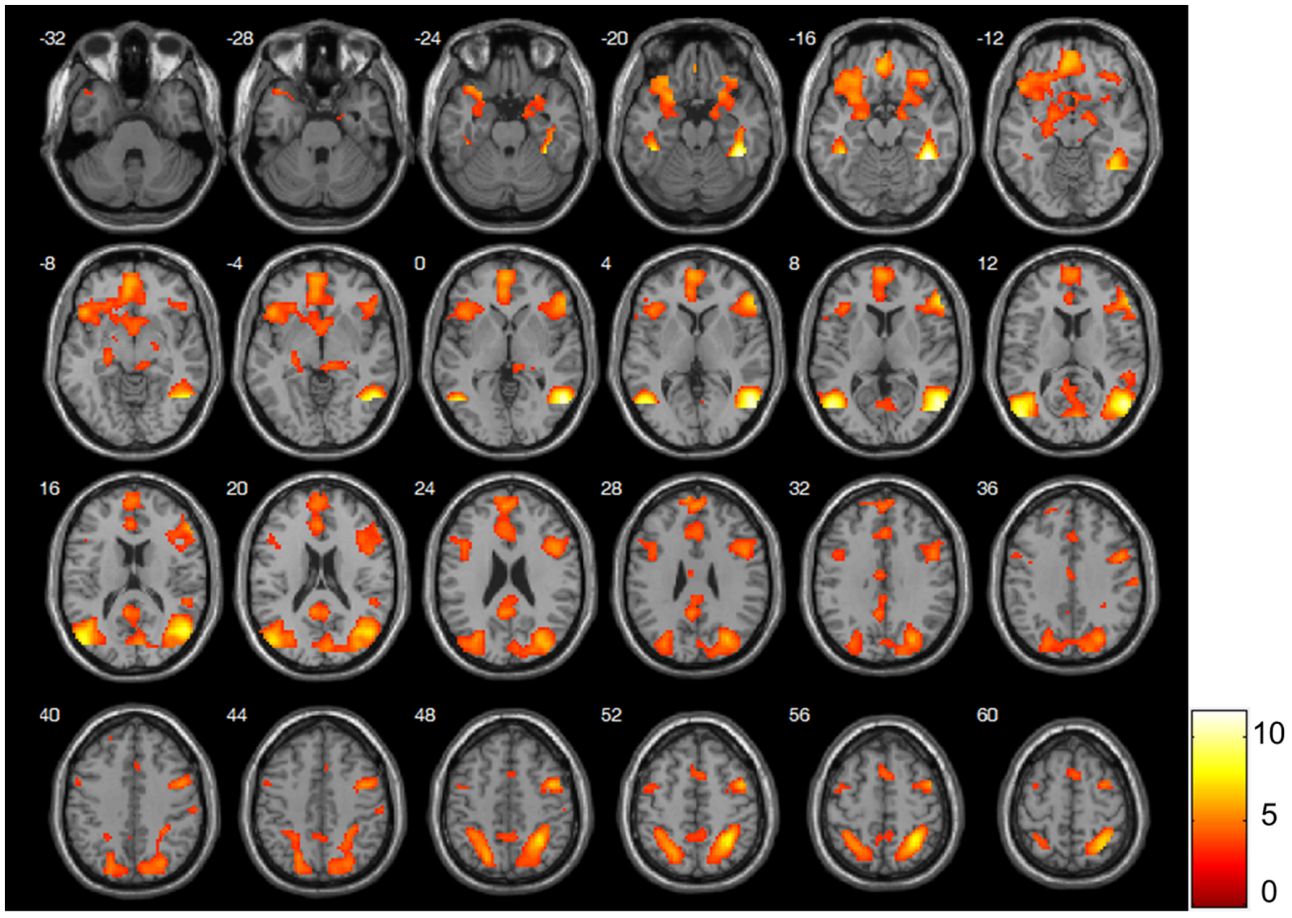
Brain activity during erotic picture processing. Images are presented in neurological convention (left is left) and with a threshold at *p*<.05 corrected. The color bar represents the *t* values applicable to the image and the numbers within the images correspond to z MNI coordinates.

During the image presentation period, cortical and subcortical brain areas showed stronger activity when participants viewed erotic pictures in contrast with neutral pictures. These areas included the OFC, medial PFC, lateral PFC, ACC, inferior temporal cortex, parietal cortex, occipital cortex, VS, amygdala, thalamus, and midbrain (see Figure 2 and Table 1 for details). Most of these areas were part of two big clusters: an anterior cluster that included frontal and limbic areas, and a posterior cluster that included occipitotemporal and parietal areas. Furthermore, a significant interaction between condition and probability was observed for activity in the bilateral precuneus (left x, y, z: −6, −67, 37; *z* = 4.09, *k* = 25; right x, y, z: 15, −58, 34; *z* = 3.93, *k* = 34). More specifically, we observed that in the erotic condition, the bilateral precuneus displayed greater activity during pictures with lower probability of appearance (Ep_50%_) than higher probability (Ep_100%_).

**Table 1:** Brain Regions Showing Increased Activity During Presentation of Erotic Images Compared With Neutral Images.

Region	Left (L) orRight (R) Side	Brodmann Area	Local Maxima Coordinates (x, y, z)	Z-Score	*k*
***Posterior Cluster***					
Fusiform Gyrus	R	20	42, −46, −17	>8	3381
Middle Temporal Gyrus	R	39	54, −64, 7	>8	
Parietal Superior	R	7	30, −52, 55	7.45	
Middle Occipital	R	19	36, −79, 22	6.03	
Parietal Superior	L	7	−21, −67, 49	5.69	
Parietal Inferior	L	40	−33, −49, 52	5.22	
Cuneus	R	18	9, −79, 16	5.20	
Posterior Cingulate	L	30	−6, −49, 19	5.06	
***Anterior Cluster***					
Inferior Frontal Cortex	R	45	54, 32, 7	6.94	3237
Lateral Prefrontal Cortex	R	6	45, 2, 52	6.18	
Orbitofrontal Cortex	R	47	36, 32, −17	5.81	
Medial Frontal Cortex	L	10	−3, 56, 1	5.79	
Insula	L	47	−24, 14, −20	5.70	
Orbitofrontal Cortex	L	47	−30, 29, −20	5.69	
Amygdala	L	–	−18, −4, −14	5.52	
Temporal Pole	L	38	−36, 20, −26	5.31	
Ventral Striatum	R	–	3, 8, −8	4.38	
***Other Clusters***					
Inferior Frontal Cortex	L	9	−42, 14, 25	4.31	175
Lateral Prefrontal Cortex	L	6	−42, −1, 55	3.75	
Fusiform Gyrus	L	20	−42, −37, −20	6.98	62
Midbrain	R	–	12, −28, −5	4.36	69
Supplementary Motor Area	–	8	0, 14, 55	4.29	87
Postcentral Gyrus	R	3	63, −19, 37	4.21	40

*Note. p*<.05 FWE corrected.

### Personality Results

To determine which dopaminergic brain areas were related to reward sensitivity during sexual stimuli processing, we calculated the correlations between SR scores and brain activity of active dopaminergic areas that yielded significant main effects during the different task conditions. For the anticipatory period, the correlations between SR scores and activity in the left insula and left OFC (i.e., the two active areas during anticipation) were not significant (Table 2).

**Table 2:** Partial Correlations Between SR Scores and Brain Activity, Removing the Effect of Age.

Contrast	Region	Left (L) orRight (R) Side	Brodmann Area	Sphere’s Center Coordinate (x, y, z)	*r*
Erotic Cues vs. Neutral Cues	Orbitofrontal Cortex	L	47	−45, 38, −14	.09
Ambiguous Cues vs. Neutral Cues	Insula	L	47	−42, 20, 1	.15
Erotic Pictures vs. Neutral Pictures	Orbitofrontal Cortex	R	47	36, 32, −17	.31*
	**Orbitofrontal Cortex**	**L**	**47**	−**30, 29,** −**20**	**.43**
	Medial Frontal Cortex	L	10	−3, 56, 1	.03
	**Insula**	**L**	**47**	−**24, 14,** −**20**	**.45**
	Midbrain	R	–	12, −28, −5	.12
	Ventral Striatum	R	–	3, 8, −8	.31*
	Amygdala	L	–	−18, −4, −14	.23

*Note.* Brain areas in bold show a significant correlation with SR scores at *p*<.05 corrected.

* Brain areas correlated with SR scores at *p*<.05 uncorrected.

**Figure 3:**
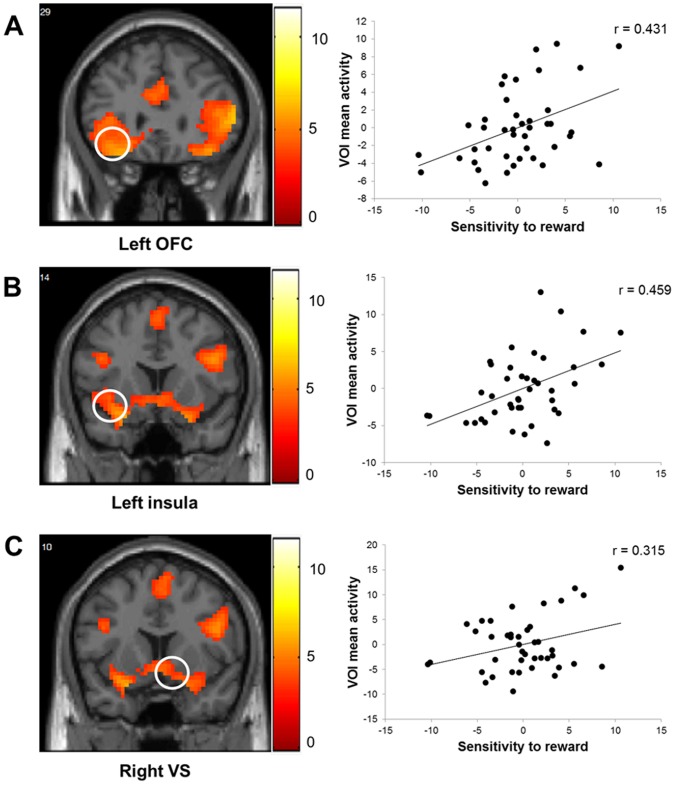
Brain areas showing positive correlation with SR scores. Left panel shows brain activity in the left orbitofrontal cortex (OFC) (a), left insula (b), and right ventral striatum (VS) (c) during erotic picture processing (*p*<.05 corrected). Images are presented in neurological convention (left is left). The color bar represents the *t* values applicable to the image and the numbers within images correspond to y MNI coordinates. Right panel shows scatterplots displaying the partial correlation between SR scores and mean VOI activity in the left OFC (a), left insula (b), and right VS (c) during erotic picture processing after removing the effects of age. The scatterplots’ axes represent the residual values from linear regressions with age as the independent variable and the other variables of interest (i.e., SR scores or mean brain activity) as the dependent variable.

For the image presentation period, we calculated the correlations between SR scores and brain activity during the processing of erotic pictures compared with neutral pictures in the bilateral OFC, left insula, medial PFC, right VS, left amygdala and midbrain. These analyses showed that SR scores positively correlated with brain activity in the left OFC (*r* = .431, *p*<.05 FWE corrected, *n* = 42; see Figure 3a) and left insula (*r* = .459, *p*<.05 FWE corrected, *n* = 42; see Figure 3b). In addition, we observed a positive correlation between SR scores and brain activity in the right VS using a lower statistical threshold of *p*<.05 uncorrected (*r* = .315, *p*<.05 uncorrected, *n* = 42; See Figure 3c). The correlations are summarized in Table 2.

## Discussion

In this study, we adapted an event-related emotional task to investigate the relationship between individual differences in reward sensitivity and brain activity during the anticipation of and reactivity to appetitive (erotic) stimuli. As expected, brain areas of the BAS showed enhanced activity during both the anticipation and presentation of erotic stimuli. Crucially, we demonstrated that activity in some of these BAS-related brain areas in response to erotic pictures was greater in individuals with stronger reward sensitivity. Thus, the results of this study were partially in consonance with the predictions of Gray’s RST since the SR scores were associated with activity in response to sexual pictures but not to cues predicting their appearance.

### Brain Activity during the Emotional Task

In order to study the different stages of sexual stimuli processing, we analyzed the picture viewing period and anticipatory period separately. Analysis of the anticipatory period yielded results consistent with our first hypothesis. As expected, we showed the involvement of BAS-related areas in the processing of erotic-anticipatory cues. By contrast, we did not find higher activity for erotic cues in comparison with ambiguous cues in BAS-related areas; thus, our second hypothesis was not supported by our data. Specifically, we demonstrated that the left lateral OFC responded to reward cues, whereas the left anterior insula responded to ambiguous cues. Recent computational models of reward processing have proposed that the lateral OFC supports flexibility by maintaining an activation-based working memory of recent reward history [42]. Deco and Rolls (2005) proposed that the OFC encodes reward rules (expectations about stimulus-contingency associations) that can be quickly reversed if expected rewards are not obtained [43]. Previous fMRI studies showed an enhanced OFC response during anticipation of reward [44], [45] and of erotic pictures in participants who were aware of contingencies between the cue and outcome [21]. On the other hand, activity in the anterior insula has been associated with anticipation of uncertain outcomes [46], [47]. Furthermore, increased anterior insula response was observed during decisions involving ambiguity when compared with those only involving risk [48]. Overall, our findings showed that lateral frontal areas are involved in the processing of erotic-anticipatory cues, suggesting a possible dissociation between the more ventral areas (i.e., OFC) involved in processing unambiguous cues and more dorsal areas (i.e., insula) involved in processing ambiguous cues. Future research should confirm this possibility.

The analysis of brain areas involved in processing erotic pictures compared with neutral pictures showed enhanced activity in the occipitotemporal cortex, parietal cortex, VS, amygdala, thalamus, midbrain, ACC, insula, lateral PFC, OFC, and medial PFC. Activity in these areas during erotic stimulation has been explained by models of sexual arousal comprising four coordinate components: cognitive, motivational, emotional, and physiological [19], [31], [49]. According to these models, activity in the occipitotemporal, parietal, and orbitofrontal areas are related to the cognitive component of sexual arousal [19], [31], [49], [50]: the evaluative process that categorizes stimuli as sexual and directs attention to them. Furthermore, activity in the insula, amygdala, rostral ACC, and medial PFC are linked to the emotional component [19], [20], [31], [50]: the processing of the subjective experience of hedonic feelings associated with sexual arousal. In addition, the rostral ACC and anterior insula constitute the physiological component of sexual arousal [19], [31]: the autonomic and endocrinological changes that lead the individual to readiness for sexual behavior. Finally, the VS, thalamus, caudal ACC, and lateral PFC embody the motivational component [19], [20], [31]: the processes that direct behavior to a sexual goal and the perceived urge to engage in sexual behavior. Although the functional interpretation of our results based on previous studies are rather speculative, we have replicated the results obtained in previous studies that associated erotic stimuli [20], [31], [49], [50], [51] with enhanced activity in brain areas involved in sexual arousal.

In addition to the study of brain areas active during erotic stimuli presentation, we performed an exploratory analysis to investigate a possible effect of interaction between condition and probability on brain activity. The result of this analysis showed that under the erotic condition, the bilateral precuneus displayed enhanced activity in response to erotic pictures with 50% probability of appearance but not those with 100% probability of appearance. A previous study found increased activity in this area during the receipt of rewards when no decision making was involved [52] while another study linked the parietal cortex to the assessment of probabilities during decision making [53]. Thus, this activity may represent evaluation of reward probability during ambiguous trials. Contrary to previous studies where reward probability was manipulated [54]–[56], we did not find activity in neither the VS nor the OFC associated with probability of erotic picture appearance. However, methodological differences may explain the discrepancy. For example, previous studies used different reward stimuli such as money or pleasant taste. Additionally, these studies employed probabilities lower than 50%, and showed a connection between higher activity in these brain areas and outcomes with lower probability of appearance [54], [56]. Thus, the 50% probability of reward used in this study may be not sufficient to generate significant differences in the activity of these brain regions.

### Correlational Effects between Personality Measure and Brain Activity

The crucial result of the present study is that reward sensitivity shows a relationship with brain activity in response to sexual stimuli presentation in brain areas related to the BAS. To be specific, participants with higher SR scores displayed enhanced activity in the left OFC, left insula, and VS while viewing erotic pictures. The association between reward sensitivity and left OFC activity may represent individual differences in the encoding of reward value. The OFC integrates sensory, affective, and motivational information to derive the value of potential reward outcomes [57]. This area has been implicated in coding the current value of stimuli [58]–[60], holding them in working memory to anticipate future consequences of behavior [42], [57]. Enhanced activity in this area has been shown during erotic reward presentation [61], whereas decreased activity has been observed after reward devaluation [62]. Thus, the increased activity in the left OFC exhibited by participants with high reward sensitivity in this study may represent their attribution of higher reward value to erotic stimuli.

The relationship between left insula activity and reward sensitivity could be related to individual differences in emotion experience. The insula has been associated with several brain functions such as the processing of interoceptive information, emotional awareness, perception of body movement, and cognitive control [63]. Previous fMRI studies on erotic stimulation showed that insula activity increases during the presentation of erotic stimuli [20], [49], [50], [51], [61], [32] under penis stimulation [64] and correlates with penis turgidity [20], [32], [65], [66]. These findings suggest the implication of the insula in monitoring interoceptive responses and are consistent with the proposed role of the insula in conscious feeling. Thus, higher insula activity in participants with high reward sensitivity may represent a stronger experience of sexual arousal in these participants. Hence, this result agrees with studies showing that participants with higher reward sensitivity display higher susceptibility to positive affect [67].

The VS is a key region of the dopaminergic reward system that is thought to be the neural substrate for individual differences in reward sensitivity [3], [68], [69]. These individual differences have been associated with the structural and functional variability of the NAcc. For example, the NAcc in individuals with high reward sensitivity shows diminished volume [24], more random resting-state neural dynamics [70], and increased response to reward-related stimuli [8]–[11]. NAcc activity in response to erotic stimuli has been related to the motivational component of sexual arousal [31]. Although of marginal statistical significance, the results of this study support increased incentive motivation attributed to participants with high reward sensitivity [3], [68], [69].

Taking these results together, we demonstrated that participants with high reward sensitivity display enhanced brain activity in areas associated with the different components of sexual arousal upon presentation of erotic pictures. These results are in line with previous research showing an association between reward sensitivity and stronger sexual arousability and excitability [12]–[17]. On the basis of these results, we may speculate that sexual arousal is at least partly mediated by BAS structures, and individual differences in reward sensitivity may modulate sexual arousal. Future research is necessary to confirm these hypotheses.

No association between reward sensitivity and brain activity regarding erotic cues was found in the analysis of the anticipatory period. Despite how the RST predicts that reward sensitivity modulates both classical and instrumental conditioning, the role of reward sensitivity in classical conditioning has been a matter of controversy [71]. Previous studies using instrumental tasks and monetary rewards have demonstrated the association between reward sensitivity and brain activity during the anticipation of reward cues [10], [11]. By contrast, to our knowledge, no study has showed a relationship between reward sensitivity and brain activity during the anticipatory period in associative tasks. Thus, the results of this study do not support our hypothesis that reward sensitivity modulates the anticipation of reward in associative conditioning, at least when presenting erotic pictures as rewards. Nevertheless, several issues must be taken into account regarding this result. First, in relation to Corr’s (2001) arguments regarding the implications of Pavlovian associations in the RST [72], the anticipatory cue of this study may be understood as a second-order association since the erotic pictures are not sexual behavior in and of themselves, which would be unconditioned stimuli. Second, it is important to note that the task procedure we used is not classical conditioning because participants were asked to make a response after each cue and after each picture in order to control their attention throughout the task. Since the objective of this study was to generalize the relationship between reward sensitivity and brain activity in response to erotic stimuli in an event-related paradigm, we adapted the task from previous studies that were not conceived to study Pavlovian conditioning. Thus, future research employing a more specific paradigm is necessary to disambiguate the role of reward sensitivity in brain activity during associative learning.

### Limitations and Future Lines of Research

Several limitations must be considered before interpreting the results of this study. First, we did not employ a physiological measure of sexual arousal (i.e., penile turgidity or heart rate) to confirm the relationship between brain activity and sexual arousal. Nevertheless, previous findings confirmed the capacity of visual sexual stimuli to generate sexual arousal [20], [32], [65], [66]. Second, the study sample is composed fully of heterosexual men. We did not include women in our study to control for sex differences in sexual processing [51] and the personality trait of reward sensitivity [23]. Thus, the results of the present study are only generalizable to men. Third, the anticipatory cues may have semantic connotations that can introduce variability depending on the individual’s experiences (i.e., “?” as a symbol of uncertainty). These cues were selected based on the paradigm of reference [22]. However, a way to avoid this problem would be to randomize the cues among participants or to use cues without any semantic connotation (i.e. fractal images), which should be taken into account in future studies and the interpretation of our results. Finally, the erotic images used in this study differed from the neutral images in terms of valence and arousal. Hence, this study does not speak to which of the factors is driving the observed results. Thus, the relationship between valence, arousal, and individual differences in brain activity in response to erotic stimuli should be addressed in future research. Additionally, given the results of this study, it would be interesting to study the influence of reward sensitivity on sexual disorders and the possible influence of reward sensitivity on women’s brain activity associated with sexual arousal.

### Conclusions

In sum, in this study, we showed that brain areas related to the BAS are engaged in the expectation and processing of erotic stimuli. We further found that individual differences in the personality trait of reward sensitivity is associated with brain activity in these areas during the processing of sexual stimuli, filling in the gap regarding the relationship between reward sensitivity and brain activation during erotic stimulus processing.
